# Inorganic pyrophosphate plasma levels in patients with GGCX-associated PXE-like phenotypes

**DOI:** 10.3389/fgene.2024.1429320

**Published:** 2024-09-27

**Authors:** Qiaoli Li, Catherine Troutman, Mary Peckiconis, Tamara Wurst, Sharon F. Terry

**Affiliations:** ^1^ Department of Biochemistry and Molecular Biology, Jefferson Institute of Molecular Medicine, Thomas Jefferson University, Philadelphia, PA, United States; ^2^ PXE International Center of Excellence in Research and Clinical Care, Thomas Jefferson University, Philadelphia, PA, United States; ^3^ PXE International, Inc., Damascus, MD, United States

**Keywords:** ABCC6, ectopic calcification, GGCX, inorganic pyrophosphate, Pseudoxanthoma elasticum

## Abstract

**Introduction:**

Pseudoxanthoma elasticum (PXE) is an autosomal recessive ectopic calcification disorder clinically affecting the skin, eyes, and vascular system. Most cases of PXE are caused by inactivating pathogenic variants in the *ABCC6* gene encoding a hepatic transmembrane efflux transporter, which facilitates the extracellular release of ATP, the precursor of inorganic pyrophosphate (PPi), a potent endogenous inhibitor of calcification. Pathogenic variants in *GGCX*, encoding γ-glutamyl carboxylase required for activation of vitamin K-dependent coagulation factors as well as matrix Gla protein (MGP) and Gla-rich protein (GRP), two inhibitors of ectopic calcification, have also been reported to cause cutaneous changes like those seen in PXE. While ectopic calcification in ABCC6 deficiency has been associated with reduced plasma levels of PPi due to loss of ABCC6 transport activity in the liver, plasma PPi levels have not been reported in patients with GGCX-associated phenotypes.

**Methods:**

We analyzed five patients from three unrelated families on their clinical, laboratory, and molecular findings who carry biallelic variants in *GGCX* and present with phenotypic characteristics associated with PXE. The variants were identified using a next-generation sequencing panel consisting of 29 genes associated with ectopic calcification.

**Results and conclusion:**

This study demonstrates that in addition to *ABCC6*, *GGCX* variants can cause the PXE phenotype, expanding PXE and perhaps other heritable ectopic calcification disorders’ clinical and genetic heterogeneity. The results also show that the plasma concentrations of PPi in these patients are not reduced compared to healthy control individuals, suggesting that plasma PPi does not govern ectopic calcification in GGCX deficiency.

## 1 Introduction

Pseudoxanthoma elasticum (PXE; OMIM #264800) is an autosomal recessive multisystem disorder clinically affecting the skin, the eyes, and the arterial blood vessels (Neldner, 1988). The phenotypic hallmark of PXE is ectopic calcium mineral deposition in non-skeletal connective tissues, particularly the elastic structures. In the skin, the primary lesions are small, yellowish papules with a predilection for flexural areas, and these lesions progressively coalesce into larger plaques of inelastic, leathery, and loose skin with a yellowish hue and loss of recoil. The characteristic eye manifestations consist of angioid streaks and peau d’orange. Calcification of the elastin-containing retinal layer, the Bruch’s membrane, causes fractures, which may eventually lead to neovascularization and retinal bleeding. Before the advent of antiangiogenesis therapies for macular bleeding, this could cause progressive loss of visual acuity. Calcification of the elastin-rich mid-laminar layer of peripheral arteries causes intermittent claudication. It occasionally results in gastrointestinal bleeding, and there is some suggestion that it might lead to hypertension and, rarely, myocardial infarction and stroke. The clinical manifestations of PXE are usually not recognized until early adulthood or adolescence, either diagnosed by finding yellowish papules of the skin that progressively coalesce to make a leathery plaque on flexor areas, the incidental finding of angioid streaks on fundoscopy in an asymptomatic individual or in a patient presenting with visual distortion secondary to subretinal hemorrhage.

PXE is usually caused by inactivating pathogenic variants in the *ABCC6* gene (Ringpfeil et al., 2000; Le Saux et al., 2000; Bergen et al., 2000). The gene encodes ABCC6, an efflux transporter protein expressed predominantly on the basolateral surface of hepatocytes in the liver and not in peripheral tissues clinically affected by PXE (Belinsky and Kruh, 1999; Scheffer et al., 2002). PXE is a metabolic disease, and recent studies have identified ABCC6 as a critical player in the generation of extracellular inorganic pyrophosphate (PPi), a potent endogenous inhibitor of calcification (Ralph et al., 2022). Specifically, ABCC6 facilitates the extracellular release of adenosine triphosphate, which is rapidly hydrolyzed by ENPP1 to generate PPi (Jansen et al., 2014; Jansen et al., 2013). As a result of nonfunctional ABCC6 transport activity, plasma PPi levels in patients with PXE and *Abcc6* knockout murine models of PXE are reduced to approximately 30%–40% of controls (Li et al., 2017; Jansen et al., 2014; Kowal et al., 2021; Saeidian et al., 2022). It is now generally accepted that deficiency of plasma PPi is the underlying cause of ectopic calcification in PXE, with low PPi levels in circulation allowing calcification of the peripheral tissue to occur. The extent of reduction of plasma PPi also correlates with the onset and disease severity in genetic and acquired conditions of ectopic calcification (Ralph et al., 2022).

Several genetically distinct clinical conditions display signs and symptoms similar to those found in PXE, with aberrant calcification of elastic structures in the skin. One such distinct condition exhibits retinal and cutaneous lesions similar to PXE with or without vitamin K-dependent coagulation factor deficiency caused by inactivating pathogenic variants in the *GGCX* gene (De Vilder et al., 2017). The gene encodes a carboxylase catalyzing the γ-glutamyl carboxylation of Gla-proteins, including clotting factors required for blood coagulation (Zhang and Ginsburg, 2004). These patients demonstrate cutaneous and retinal lesions that are similar and, in some cases, indistinguishable from those found in PXE. While PPi is a strong determinant of ectopic calcification in several genetic and acquired diseases of ectopic calcification (Ralph et al., 2022), the plasma PPi concentrations in these phenotypes apparently caused by pathogenic variants in GGCX have not been reported. It is unknown if plasma PPi deficiency would also contribute to ectopic calcification in GGCX deficiency.

This study examined five affected individuals from three unrelated families with phenotypes consistent with PXE, with or without vitamin K-dependent coagulation factor deficiency, who were negative for variants in *ABCC6* but carried biallelic pathogenic or likely pathogenic variants in the *GGCX* gene. The aim of this study was to measure concentrations of PPi in these patients to better understand the pathogenesis of ectopic calcification in this PXE-like disorder.

## 2 Materials and methods

### 2.1 Study participants

All patients were enrolled with written informed consent into this study with approval from the Genetic Alliance Institutional Review Board (Approval number PXE001). These patients have a clinical diagnosis of PXE confirmed by a dermatologist and/or an ophthalmologist, based on the diagnostic criteria proposed by the PXE International Research Consortium at the 2014 Annual Meeting (Uitto et al., 2014). They had either a skin biopsy, which by histopathological stains revealed fragmented elastic fibers and calcium phosphate deposition in the lesional skin, and/or retinal changes, including peau d’orange and angioid streaks. Patients were registered in the databases of PXE International, an advocacy organization for PXE. We used the Phenodex score, a severity index created by PXE International and adopted internationally to assess phenotypes in the five common organ systems associated with PXE: skin (S), eyes (E), cardiac (C), vasculature (V), and gastrointestinal (GI), to determine the clinical severity of PXE (Pfendner et al., 2007).

### 2.2 Variant detection and bioinformatics

Genomic DNA was obtained from PXE International Biobank, extracted from saliva (Qiagen, Valencia, CA), or from peripheral blood samples (DNA Genotek Inc., Ontario, Canada) of affected individuals. Variant detection was performed using a next-generation sequencing (NGS) panel containing 29 ectopic mineralization-associated genes, including *ABCC6* and *GGCX* (Saeidian et al., 2022). The panel includes *ABCC6, ADIPOQ, AHSG, ANKH, APOE, ATF4, CASR, ENPP1, FAM20A, FGF23, GALNT3, GGCX, KL, MGP, NT5E, SAMD9, SLC20A2, SLC29A1, SPP1, TRIM24, TNFRSF11B*, *A2AP, ALPL, ENTPD1, PAI-1, PLAT, PLAU, PLAUR,* and *PLG*. The TruSeq Custom Amplicon kit (Illumina Inc., San Diego, CA) and DesignStudio (Illumina, Inc., San Diego, CA) were used for target enrichment and library design, respectively. All coding exons, at least 20 bp of the intron at each intron-exon boundary, and up to 50 bp of 3′- and 5′-untranslated regions were targeted. The library had 649 amplicon probes with an average product size of 200 bp, covering 99% of targeted bases. The variants were subsequently validated by Sanger sequencing.

*GGCX* variant nomenclature was based on NM_000821.7. The variant nomenclature followed the recommendations of the Human Genome Variation Society (http://www.hgvs.org/mutnomen/). The number of individuals carrying the specific variant as homozygous and the minor allele frequency in the general population was extracted from the Genome Aggregation Database (gnomAD) (gnomad.broadinstitute.org) and BRAVO (https://bravo.sph.umich.edu/freeze8/hg38/) consisting of over 120,000 and 150,000 apparently healthy individuals, respectively. Various *in silico* prediction programs and the Combined Annotation Depletion (CADD) score were used to assess the effects of variants on the protein function (Kircher et al., 2014; Rentzsch et al., 2019). Classification of variants follows the latest guidelines of ClinGen and the American College of Medical Genetics and Genomics/Association for Molecular Pathology (ACMG/AMP), which classifies variants as benign (B), likely benign (LB), variants of unknown significance (VUS), likely pathogenic (LP), and pathogenic (P) (Nykamp et al., 2017; Richards et al., 2015).

### 2.3 Plasma PPi quantification

Whole blood was collected into CTAD (citrate, theophylline, adenosine, and dipyridamole) and transferred immediately to EDTA tubes (BD Diagnostics, Franklin Lakes, NJ). Plasma was collected after centrifugation by 2,000 g for 15 min at 4°C. As platelets are rich with PPi, the plasma was depleted of platelets by filtration through a Centrisart I 300-kDa mass cutoff filter (Sartorius, New York, NY) and stored at −80°C until analysis. The concentration of PPi in platelet-free plasma was measured by an enzymatic reaction using ATP sulfurylase (MCLAB, South San Francisco, CA) to convert PPi into ATP in the presence of excess adenosine 5′phosphosulfate (Cayman Chemical Company, Ann Arbor, Michigan), as described previously (Kowal et al., 2021; Saeidian et al., 2022).

### 2.4 Statistical analysis

Statistical analyses were performed using ordinary one-way ANOVA. Statistical significance was considered with *P* < 0.05. All statistical analyses were completed using Prism 9 (GraphPad, San Diego, CA).

## 3 Results

### 3.1 Clinical features of patients with PXE-like phenotypes

In this study, we identified five subjects from three unrelated families with confirmed diagnoses of PXE.

**FIGURE 1:**
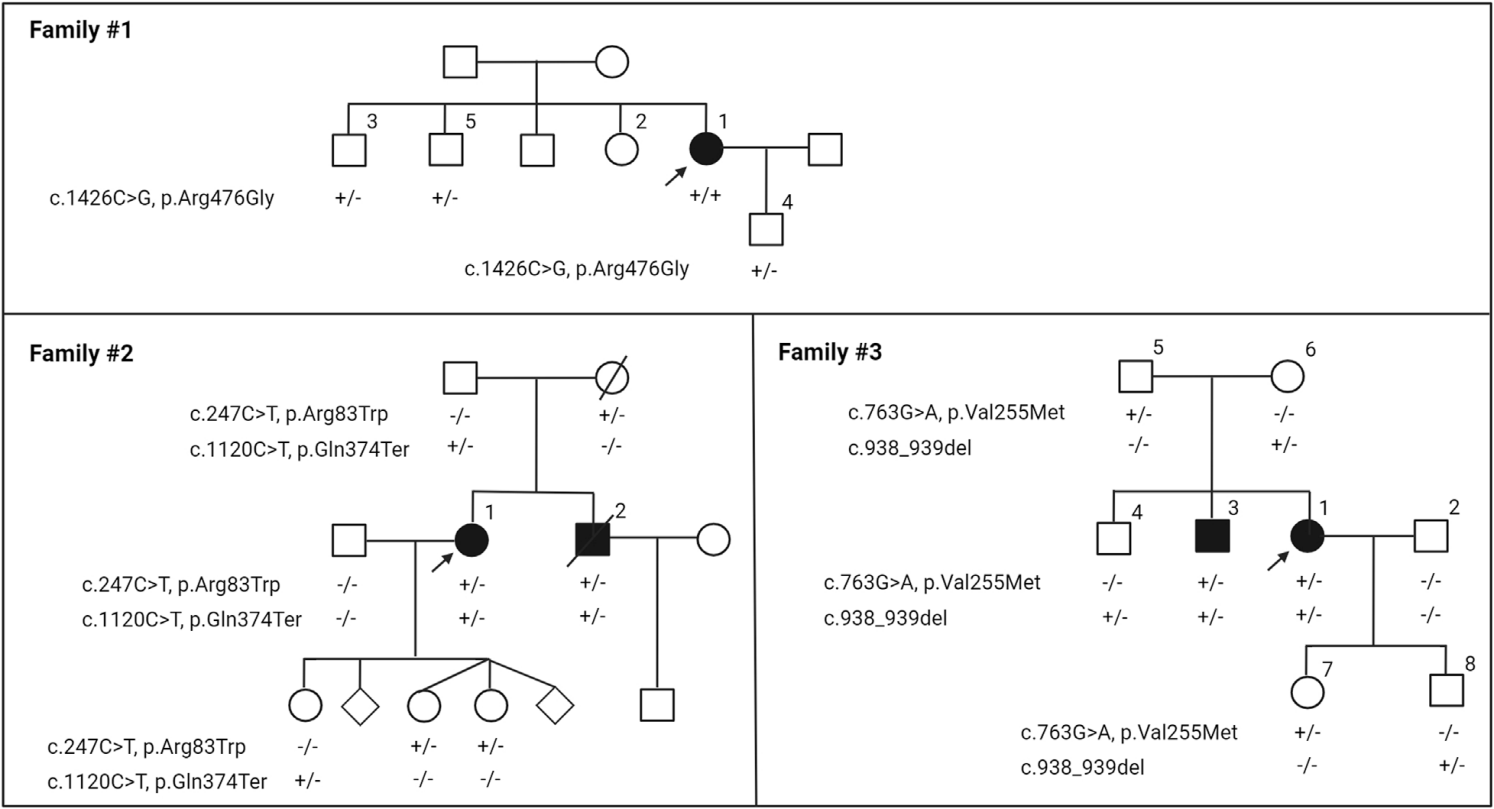
Nuclear pedigrees of families with a diagnosis of PXE but carrying biallelic variants in the *GGCX* gene. The patient identifiers were placed above their symbol. The variants identified in the *GGCX* gene in the family members are indicated below each individual: +/+, variants present in both alleles; +/−, heterozygous; −/−, homozygous for the wild-type allele. Arrows indicate the proband in each family.

Family #1 has one affected individual of Indian descent, patient 1, a female aged 57 years at the time of this writing (Figure 1). At age 19, she noticed lesions, papules, and loose skin on her neck, axilla, antecubital fosse, and inguinal region. A dermatologist diagnosed her with PXE when she was 32. At age 33, she was diagnosed with angioid streaks in both eyes, but has not progressed to have any retinal neovascularization or bleeding. Her Phenodex scores (an index specific to detailing the common manifestations of PXE) are reported in Supplementary Table S1.

Family #2 has two affected individuals of Irish descent, 1 and 2 (Figure 1). The proband 1 is a 65-year-old female who was initially diagnosed with an autosomal recessive disorder of vitamin K-dependent coagulation factor deficiency at age 13. Her bleeding disorder was extensively studied, and has been reported previously (Goldsmith et al., 1982). At the time of her initial hematological evaluation, both prothrombin and factor X activities were severely reduced: prothrombin time (s) 17.8–20.3 (normal range12–16), prothrombin (% normal) 20 (normal limits 65–150), factor X (% normal) 20%–22% (normal range 65–185). She responded to 5 mg vitamin K1 administered daily without further significant bleeding problems. The proband had a younger brother, 2, who also had a similar bleeding diathesis with similar laboratory findings.

**FIGURE 2:**
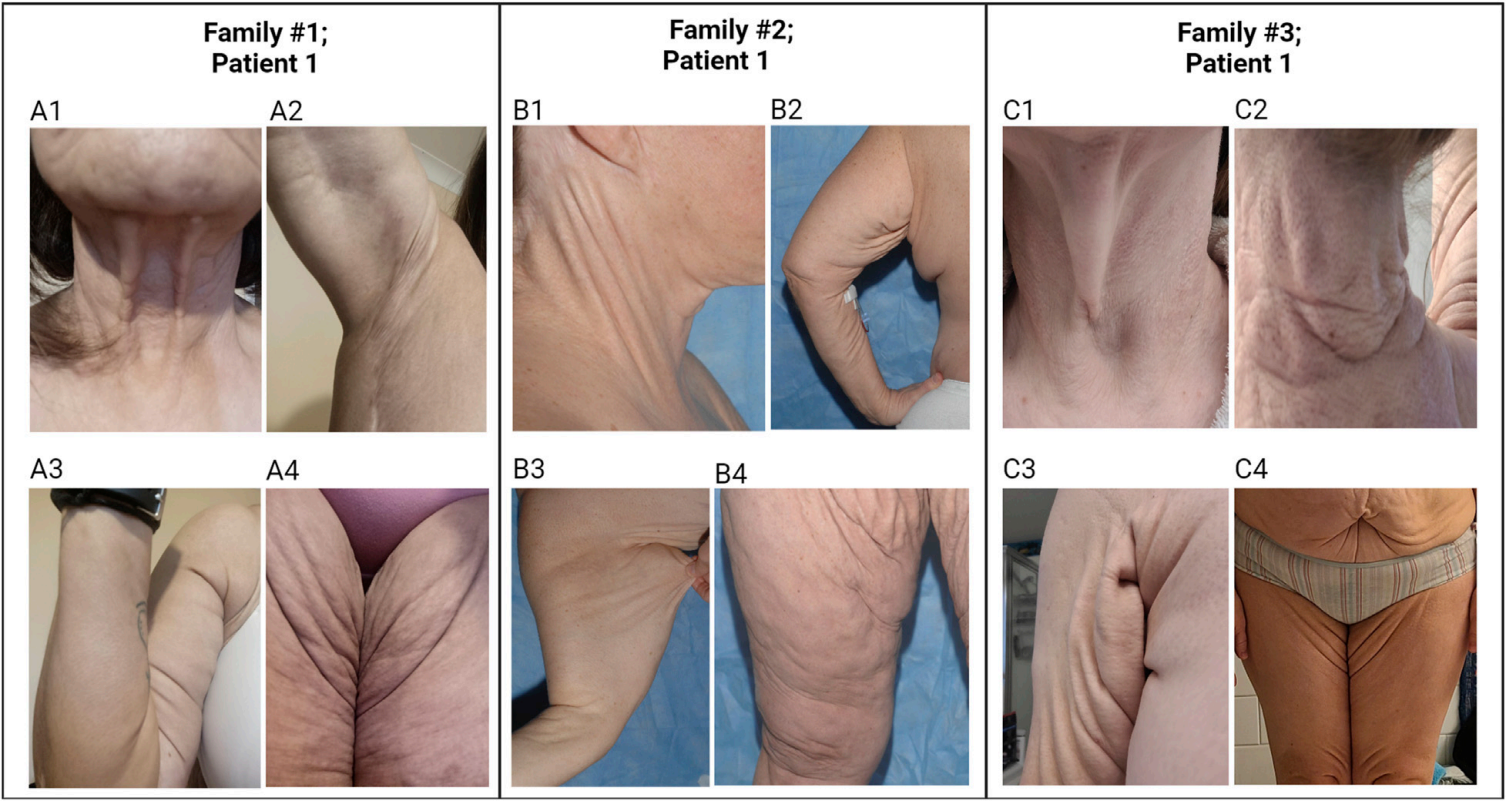
Cutaneous features in individuals with PXE-like phenotypes. Patient 1 in Family #1: The patient’s skin is loose and redundant in the anterior neck (**A1**), axillary areas (**A2, A3**), and groin areas (**A4**). Patient 1 in Family #2: The patient’s skin is loose and sagging in the lateral neck (**B1**), axillary areas (**B2, B3**), and groin areas (**B4**). Patient 1 in Family #3: The patient’s skin is loose and sagging in the lateral neck (**C1**), posterior neck (**C2**), axillary area (**C3**), and abdomen and groin areas (**C4**). The yellowish papules and plaques in the skin of these patients demonstrate characteristic features of PXE. Note that pictures in **(B1, B4)** were published previously (Li et al., 2009b).

At age 20, patient 1 was evaluated by dermatologists for cutaneous changes and she was diagnosed with PXE. At age 49, the proband exhibited loose, sagging, and redundant skin with loss of recoil, particularly affecting the neck, axillary, and groin areas (Figure 2). Her physical examinations also revealed small yellowish papules coalescing into larger plaques in the flexor areas; these lesions demonstrated characteristic features of PXE. At age 30, the proband’s ophthalmologist reported evidence of very faint angioid streaks and no choroidal neovascularization. At the time of this writing, her angioid streaks are still faint and there have been no retinal bleeds. The proband also complained of intermittent claudication. She had extensive plastic surgery including her neck, face, axilla, and abdomen. The brother of the proband also had co-existent PXE and vitamin K-dependent coagulation factor deficiency. It is unknown it he had peau d’orange or angioid streaks. He had no retinal bleeds or vision loss at the time of his death, at age 55 of an abdominal aortic aneurysm. The dermatological and ophthalmological features of this family were reported previously (Li et al., 2009b). Their skin, eyes, and vascular systems are scored using the Phenodex (Supplementary Table S1).

Family #3 had two affected subjects of Irish/German descent, 1 and 3 (Figure 1). The proband 1 is a female aged 45 years. Around age 13, she noticed lesions, papules, and loose skin on her axillae, neck, and under the chin. In her 20 s, the loose and sagging skin spread to other areas of the skin, and she reports it is now severe in all areas. Histopathology of the lesional skin revealed calcification in the dermis, and a dermatologist diagnosed her with PXE at age 26. By age 35, she had sagging skin in her anterior and posterior neck, axillary, and inguinal areas (Figure 2). At the age of 26 years, the ophthalmologist reported faint angioid streaks in both eyes, and an unspecified “retinopathy” in the periphery of both eyes. At age 40, she was diagnosed with Grade 1 mitral valve prolapse. The proband’s brother 3 noticed similar skin changes at age 22 and was diagnosed with PXE at age 31. The other organs were not affected except for skin manifestations (Supplementary Table S1). There was no evidence of blood clotting problems for either individual.

### 3.2 Identification of *GGCX* variants

The proband 1 in Family #1 had a homozygous c.1426C>G, p.Arg476Gly variant in the *GGCX* gene (Figure 1). This variant was not previously described. Two of her siblings and her son are heterozygotes of this variant and clinically healthy. Patients 1 and 2 in Family #2 were compound heterozygous for c.247C>T, p.Arg83Trp and c.1120C>T, p.Gln374Ter in the *GGCX* gene (Figure 1). The proband’s parents and three children are heterozygotes. Both variants have been previously reported (Li et al., 2009b). Patients 1 and 3 in Family #3 were compound heterozygous for c.763G>A, p.Val255Met and c.938_939del in *GGCX* (Figure 1). The p.Val255Met variant was previously reported (Li et al., 2009a) and the c.938_939del variant was reported in ClinVar. Parents and an older sibling are heterozygotes. This inheritance is consistent with autosomal recessive mode. No pathogenic sequence variations were found in these individuals in other genes in the panel, including *ABCC6*.

### 3.3 Bioinformatics analyses of *GGCX* variants

Five *GGCX* variants were identified in three families (Table 1). These variants are rare, with minor allele frequencies lower than 0.015% in the general population genetics databases – gnomAD and BRAVO. The aggregated predictions were deleterious for four of them: c.1426C>G (p.Arg476Gly), c.247C>T (p.Arg83Trp), c.1120C>T (p.Gln374Ter), and c.763G>A (p.Val255Met) While the aggregated prediction was benign for c.938_939del, individual prediction algorithms such as Mutation Taster, PolyPhen2, and SIFT predict it as disease-causing. The ClinGen and American College of Medical Genetics and Genomics/Association for Molecular Pathology (ACMG/AMP) classifies all variants as likely pathogenic (LP) or pathogenic (P). All variants had CADD scores above 20, predicted to be among the top 1% most deleterious to the human genome.

**TABLE 1:** Bioinformatics analyses of *GGCX* variant**s**.

Variant^a^	Domain	ClinVar ID	Population data No. of homozygous; MAF (%)		Prediction outcome by			
			gnomAD (Aggregated)^b^	BRAVO^c^	Aggregated outcome^d^	ACMG/AMP^e^	ClinGen^f^	CADD score^g^
c.1426C>G, p.Arg476Gly	ER lumen	3335889	0; 0.00068	0; 0.0020	Deleterious	LP	LP	34.00
c.247C>T, p.Arg83Trp	ER lumen	2203111	0; 0.0025	0; 0.0023	Deleterious	P	P	23.70
c.1120C>T, p.Gln374Ter	Membrane	16202	—	—	Deleterious	P	P	40.00
c.763G>A, p.Val255Met	ER lumen	16206	0; 0.0139	0; 0.0050	Deleterious	LP	LP	25.70
c.938_939del, p.Pro313Argfs*33	Membrane	3023945	0; 0.0060	0; 0.0066	Benign	P	P	29.1

^a^Variant nomenclature was based on NM_000821.7.

^b^The Genome Aggregation Database (gnomAD v4.1) consists of over 800,000 unrelated individuals sequenced in population genetic studies. The number of individuals carrying the specific variant as homozygous and the minor allele frequency (MAF) are provided.

^c^BRAVO, is a variant browser containing allele frequencies for 868 million variants observed in 150,899 deeply sequenced genomes from the TOPMed, data Freeze 18.

^d^While there are over ten different prediction algorithms, only the aggregated outcome is provided (https://franklin.genoox.com/clinical-db/home).

^e^The American College of Medical Genetics and Genomics/Association for Molecular Pathology (ACMG/AMP) classifies variants as benign (B), likely benign (LB), VUS (variants of unknown significance), likely pathogenic (LP), and pathogenic (P).

^f^ClinGen is a National Institutes of Health (NIH)-funded resource dedicated to building a central resource that defines the clinical relevance of genes and variants for use in precision medicine and research. ClinGen variant pathogenicity curation utilizes the ACMG/AMP, guideline for sequence variant interpretation.

^g^The Combined Annotation Depletion score (CADD) is provided for each variant. CADD, score 20 of a variant suggests top 1% most deleterious to the human genome.

### 3.4 Circulating concentrations of PPi in PXE-like patients carrying biallelic *GGCX* variants

**FIGURE 3:**
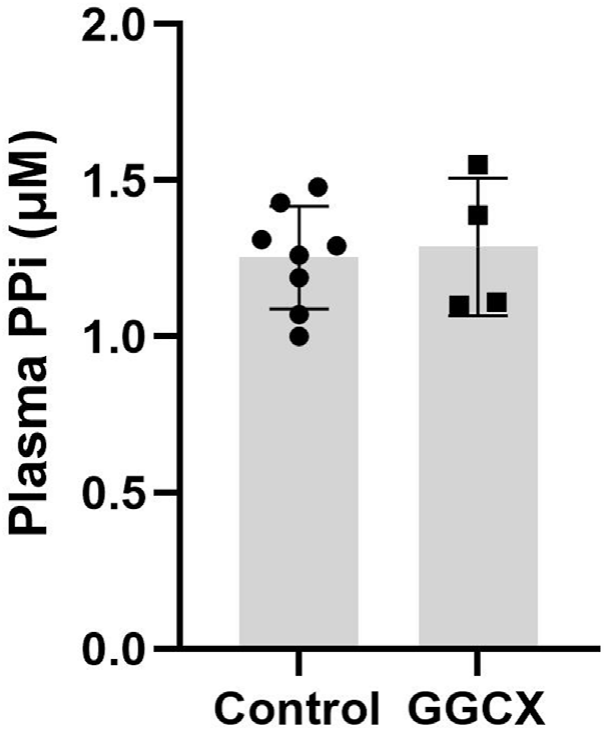
Plasma PPi concentrations in individuals with GGCX-associated PXE-like phenotypes. Individuals with biallelic *GGCX* variants had PPi plasma concentrations indistinguishable from healthy control individuals. The physiologic PPi plasma concentrations were obtained from 8 healthy control volunteers (mixed male and female individuals in the age range of 49–65 years of age). No statistical difference was found between the two groups. Data represent mean ± SD.

Plasma PPi concentrations were determined in patient 1 in Family #1, patient 1 in Family #2, patients 1 and 3 in Family #3. These individuals harbor biallelic pathogenic variants in *GGCX*. The results demonstrated that these individuals, despite the diagnosis of PXE and being registered in PXE International, had PPi plasma concentrations indistinguishable from healthy controls (*p* > 0.5) (Figure 3).

## 4 Discussion

Considering the genetic heterogeneity of heritable ectopic calcification disorders, our variant detection employed a next-generation sequencing panel consisting of 29 genes associated with ectopic calcification (Saeidian et al., 2022). The gene panel includes *ABCC6*, the gene discovered to be associated with most cases of PXE in 2000 (Ringpfeil et al., 2000; Le Saux et al., 2000; Bergen et al., 2000). The panel also has *GGCX*, whose pathogenic variants were first linked to multiple vitamin K-dependent clotting factor deficiency (VKCFD1; OMIM #277450), an autosomal recessive disorder characterized by a mild to severe bleeding tendency and a predisposition to thrombotic events (Brenner et al., 1998). Recently, variants in *GGCX* were associated with a new clinical entity including some similarities to the PXE phenotype (catalogued as “PXE-like”) and also with multiple coagulation factor deficiency (PXE/VKCFD1; OMIM #610842) (Vanakker et al., 2007). All five subjects in the study were clinically diagnosed with PXE and referred by PXE International. The sequencing panel failed to detect pathogenic variants in *ABCC6*. Instead, we identified biallelic variants in the *GGCX* gene in all five individuals. We also identified a previously unreported *GGCX* variant, c.1426C>G, p.Arg476Gly. While p.Arg476Gly is new, two variants at the same amino acid, c.1426C>T, p.Arg476Cys, and c.1427G>A, p.Arg476His, have been previously reported in patients with phenotypes similar to PXE (Vanakker et al., 2007; Watzka et al., 2014). Functional studies of the variant proteins at the same amino acid, p.Arg476Cys and p.Arg476His, demonstrate abolished carboxylation of vitamin K-dependent proteins, which is critical for blood coagulation, vascular calcification, and bone metabolism (Ghosh et al., 2021; Hao et al., 2021). The c.938_939del variant is expected to cause an out-of-frame translation followed by a premature termination codon (p.Pro313Argfs*33), resulting in a truncated and non-functional GGCX protein. All the variants are rare, with minor allele frequencies lower than 0.015% in the general population.

The *GGCX* gene encodes a carboxylase required for γ-glutamyl carboxylation of Gla-proteins, including clotting factors in the liver, enabling them to control blood coagulation (Zhang and Ginsburg, 2004; Berkner and Runge, 2022). Thus, γ-glutamyl carboxylase deficiency explains the coagulation disorder. In our study, Family #2 exhibits two discrete phenotypic features: a co-existence of deficiency of vitamin K-dependent coagulation factors and cutaneous features similar to the abnormal skin elasticity, skin laxity, and ectopic skin calcification of PXE. However, Family #1 and #3 have what appears to be a far more severe cutaneous PXE phenotype without bleeding issues. The types of variants in the *GGCX* gene influence the severity of the coagulopathy. This notion is reflected by observations that not all patients with *GGCX* variants have vitamin K-dependent coagulation factor deficiency (De Vilder et al., 2017), which functional studies could explain that distinct variants in the *GGCX* gene can reduce the corresponding carboxylase activity to a different degree in different substrates (Tie et al., 2016; Hao et al., 2021; Rishavy et al., 2022; Ghosh et al., 2021).

While plasma PPi concentrations in PXE patients with ABCC6 deficiency were reduced to approximately 30%–40% of controls, we found that patients with GGCX deficiency had similar plasma PPi levels to controls. The results point to different pathomechanisms of ectopic calcification in these genetic diseases caused by variants in distinct genes. ABCC6 is a hepatic efflux transporter mediating the extracellular release of ATP, which is cleaved to PPi to prevent ectopic calcification. The current prevailing view for the development of ectopic calcification in patients with GGCX deficiency relies on the ability of matrix Gla protein (MGP) and Gla-rich protein (GRP) to prevent premature calcification when modified by γ-glutamyl carboxylation (Ghosh et al., 2022; De Vilder et al., 2017). If the carboxylation reaction does not occur due to deficient carboxylase enzyme activity, the undercarboxylated forms of MGP and GRP are inactive and cannot prevent ectopic calcification.

In conclusion, we identified five individuals who carry *GGCX* variants and develop signs and symptoms similar to PXE, expanding the *GGCX* variant landscape and genetic spectrum responsible for PXE. Despite their associated gene being *GGCX*, all five individuals in the study were diagnosed through a positive von Kossa stain and either peau d’orange or angioid streaks, hallmarks of the PXE diagnostic criteria. They all receive support from PXE International. The ectopic calcification in patients with GGCX deficiency cannot be explained by low plasma concentrations of PPi. The results suggest that although PPi is a significant determinant of ectopic calcification, it does not govern ectopic calcification in the context of GGCX deficiency. One of the limitations of the study relates to the small number of individuals analyzed. Further work is necessary to examine PPi homeostasis in patients with GGCX deficiency.

## Data Availability

The previously unreported GGCX variant, c.1426C>G, p.Arg476Gly, presented in the study, is deposited in ClinVar, accession number SCV005092011.1.

## References

[B1] Belinsky M. G. Kruh G. D. (1999). MOAT-E (ARA) is a full-length MRP/cMOAT subfamily transporter expressed in kidney and liver. Br. J. Cancer 80, 1342–1349. 10.1038/sj.bjc.6690527 10424734 PMC2363063

[B2] Bergen A. A. Plomp A. S. Schuurman E. J. Terry S. Breuning M. Dauwerse H. (2000). Mutations in ABCC6 cause pseudoxanthoma elasticum. Nat. Genet. 25, 228–231. 10.1038/76109 10835643

[B3] Berkner K. L. Runge K. W. (2022). Vitamin K-dependent protein activation: normal gamma-glutamyl carboxylation and disruption in disease. Int. J. Mol. Sci. 23, 5759. 10.3390/ijms23105759 35628569 PMC9146348

[B4] Brenner B. Sanchez-Vega B. Wu S. M. Lanir N. Stafford D. W. Solera J. (1998). A missense mutation in gamma-glutamyl carboxylase gene causes combined deficiency of all vitamin K-dependent blood coagulation factors. Blood 92, 4554–4559. 10.1182/blood.v92.12.4554 9845520

[B5] De Vilder E. Y. Debacker J. Vanakker O. M. (2017). GGCX-associated phenotypes: an overview in search of genotype-phenotype correlations. Int. J. Mol. Sci. 18, 240. 10.3390/ijms18020240 28125048 PMC5343777

[B6] Ghosh S. Kraus K. Biswas A. Muller J. Buhl A. L. Forin F. (2021). GGCX mutations show different responses to vitamin K thereby determining the severity of the hemorrhagic phenotype in VKCFD1 patients. J. Thromb. Haemost. 19, 1412–1424. 10.1111/jth.15238 33590680

[B7] Ghosh S. Oldenburg J. Czogalla-Nitsche K. J. (2022). The role of GRP and MGP in the development of non-hemorrhagic VKCFD1 phenotypes. Int. J. Mol. Sci. 23, 798. 10.3390/ijms23020798 35054981 PMC8775833

[B8] Goldsmith G. H. JR. Pence R. E. Ratnoff O. D. Adelstein D. J. Furie B. (1982). Studies on a family with combined functional deficiencies of vitamin K-dependent coagulation factors. J. Clin. Invest 69, 1253–1260. 10.1172/jci110564 7085873 PMC370197

[B9] Hao Z. Jin D. Y. Chen X. Schurgers L. J. Stafford D. W. Tie J. K. (2021). γ-Glutamyl carboxylase mutations differentially affect the biological function of vitamin K-dependent proteins. Blood 137, 533–543. 10.1182/blood.2020006329 33507293 PMC7845004

[B10] Jansen R. S. Duijst S. Mahakena S. Sommer D. Szeri F. Varadi A. (2014). ABCC6-mediated ATP secretion by the liver is the main source of the mineralization inhibitor inorganic pyrophosphate in the systemic circulation-brief report. Arterioscler. Thromb. Vasc. Biol. 34, 1985–1989. 10.1161/ATVBAHA.114.304017 24969777 PMC6743317

[B11] Jansen R. S. Kucukosmanoglu A. De Haas M. Sapthu S. Otero J. A. Hegman I. E. (2013). ABCC6 prevents ectopic mineralization seen in pseudoxanthoma elasticum by inducing cellular nucleotide release. Proc. Natl. Acad. Sci. U. S. A. 110, 20206–20211. 10.1073/pnas.1319582110 24277820 PMC3864344

[B12] Kircher M. Witten D. M. Jain P. O'Roak B. J. Cooper G. M. Shendure J. (2014). A general framework for estimating the relative pathogenicity of human genetic variants. Nat. Genet. 46, 310–315. 10.1038/ng.2892 24487276 PMC3992975

[B13] Kowal L. Huang J. Luo H. Singh J. Snook A. E. Uitto J. (2021). Functional assessment of missense variants in the ABCC6 gene implicated in pseudoxanthoma elasticum, a heritable ectopic mineralization disorder. J. Invest Dermatol 142, 1085–1093. 10.1016/j.jid.2021.08.435 34597610 PMC8957506

[B14] LE Saux O. Urban Z. Tschuch C. Csiszar K. Bacchelli B. Quaglino D. (2000). Mutations in a gene encoding an ABC transporter cause pseudoxanthoma elasticum. Nat. Genet. 25, 223–227. 10.1038/76102 10835642

[B15] Li Q. Grange D. K. Armstrong N. L. Whelan A. J. Hurley M. Y. Rishavy M. A. (2009a). Mutations in the GGCX and ABCC6 genes in a family with pseudoxanthoma elasticum-like phenotypes. J. Invest Dermatol 129, 553–563. 10.1038/jid.2008.271 18800149 PMC2900916

[B16] Li Q. Kingman J. VAN De Wetering K. Tannouri S. Sundberg J. P. Uitto J. (2017). Abcc6 knockout rat model highlights the role of liver in PPi homeostasis in pseudoxanthoma elasticum. J. Invest Dermatol 137, 1025–1032. 10.1016/j.jid.2016.11.042 28111129 PMC6251403

[B17] Li Q. Schurgers L. J. Smith A. C. Tsokos M. Uitto J. Cowen E. W. (2009b). Co-existent pseudoxanthoma elasticum and vitamin K-dependent coagulation factor deficiency: compound heterozygosity for mutations in the GGCX gene. Am. J. Pathol. 174, 534–540. 10.2353/ajpath.2009.080865 19116367 PMC2630561

[B18] Neldner K. H. (1988). Pseudoxanthoma elasticum. Clin. Dermatol 6, 1–159. 10.1016/0738-081x(88)90003-x 3359381

[B19] Nykamp K. Anderson M. Powers M. Garcia J. Herrera B. Ho Y. Y. (2017). Sherloc: a comprehensive refinement of the ACMG-AMP variant classification criteria. Genet. Med. 19, 1105–1117. 10.1038/gim.2017.37 28492532 PMC5632818

[B20] Pfendner E. G. Vanakker O. M. Terry S. F. Vourthis S. Mcandrew P. E. Mcclain M. R. (2007). Mutation detection in the ABCC6 gene and genotype-phenotype analysis in a large international case series affected by pseudoxanthoma elasticum. J. Med. Genet. 44, 621–628. 10.1136/jmg.2007.051094 17617515 PMC2597973

[B21] Ralph D. VAN De Wetering K. Uitto J. Li Q. (2022). Inorganic pyrophosphate deficiency syndromes and potential treatments for pathologic tissue calcification. Am. J. Pathol. 192, 762–770. 10.1016/j.ajpath.2022.01.012 35182493 PMC9088198

[B22] Rentzsch P. Witten D. Cooper G. M. Shendure J. Kircher M. (2019). CADD: predicting the deleteriousness of variants throughout the human genome. Nucleic Acids Res. 47, D886–D894. 10.1093/nar/gky1016 30371827 PMC6323892

[B23] Richards S. Aziz N. Bale S. Bick D. Das S. Gastier-Foster J. (2015). Standards and guidelines for the interpretation of sequence variants: a joint consensus recommendation of the American College of medical genetics and Genomics and the association for molecular Pathology. Genet. Med. 17, 405–424. 10.1038/gim.2015.30 25741868 PMC4544753

[B24] Ringpfeil F. Lebwohl M. G. Christiano A. M. Uitto J. (2000). Pseudoxanthoma elasticum: mutations in the MRP6 gene encoding a transmembrane ATP-binding cassette (ABC) transporter. Proc. Natl. Acad. Sci. U. S. A. 97, 6001–6006. 10.1073/pnas.100041297 10811882 PMC18548

[B25] Rishavy M. A. Hallgren K. W. Wilson L. A. Hiznay J. M. Runge K. W. Berkner K. L. (2022). GGCX mutants that impair hemostasis reveal the importance of processivity and full carboxylation to VKD protein function. Blood 140, 1710–1722. 10.1182/blood.2021014275 35767717 PMC9707401

[B26] Saeidian A. H. Youssefian L. Huang J. Touati A. Vahidnezhad H. Kowal L. (2022). Genetic heterogeneity of heritable ectopic mineralization disorders in a large international cohort. Genet. Med. 24, 75–86. 10.1016/j.gim.2021.08.011 34906475 PMC8943706

[B27] Scheffer G. L. Hu X. Pijnenborg A. C. Wijnholds J. Bergen A. A. Scheper R. J. (2002). MRP6 (ABCC6) detection in normal human tissues and tumors. Lab. Invest 82, 515–518. 10.1038/labinvest.3780444 11950908

[B28] Tie J. K. Carneiro J. D. Jin D. Y. Martinhago C. D. Vermeer C. Stafford D. W. (2016). Characterization of vitamin K-dependent carboxylase mutations that cause bleeding and nonbleeding disorders. Blood 127, 1847–1855. 10.1182/blood-2015-10-677633 26758921 PMC4832504

[B29] Uitto J. Jiang Q. Varadi A. Bercovitch L. G. Terry S. F. (2014). Pseudoxanthoma elasticum: diagnostic features, classification, and treatment options. Expert Opin. Orphan Drugs 2, 567–577. 10.1517/21678707.2014.908702 25383264 PMC4219573

[B30] Vanakker O. M. Martin L. Gheduzzi D. Leroy B. P. Loeys B. L. Guerci V. I. (2007). Pseudoxanthoma elasticum-like phenotype with cutis laxa and multiple coagulation factor deficiency represents a separate genetic entity. J. Invest Dermatol 127, 581–587. 10.1038/sj.jid.5700610 17110937

[B31] Watzka M. Geisen C. Scheer M. Wieland R. Wiegering V. Dorner T. (2014). Bleeding and non-bleeding phenotypes in patients with GGCX gene mutations. Thromb. Res. 134, 856–865. 10.1016/j.thromres.2014.07.004 25151188

[B32] Zhang B. Ginsburg D. (2004). Familial multiple coagulation factor deficiencies: new biologic insight from rare genetic bleeding disorders. J. Thromb. Haemost. 2, 1564–1572. 10.1111/j.1538-7836.2004.00857.x 15333032

