# Supplementary material for: Inorganic pyrophosphate plasma levels in patients with GGCX-associated PXE-like phenotypes
Source: Front Genet. 2024 Sep 27;15:1429320. doi: 10.3389/fgene.2024.1429320 (PMC11466855; doi:10.3389/fgene.2024.1429320)
Supplement: Supplementary file 1 [file Table1.DOCX]

**Table S1. Phenodex scores of patients diagnosed with PXE**

| **Family #** | **Patient #** | **Age (years)** | **Phenodex scores*** | | | | |
| --- | --- | --- | --- | --- | --- | --- | --- |
|  |  |  | Skin (S) | Eye (E) | Cardiac (C) | Vascular (V) | Gastrointestinal (GI) |
| 1 | 001 | 35 | 3 | 2 | 0 | 2 | 0 |
|  |  | 57 | 3 | 2 | 0 | 2 | 0 |
| 2 | 001 | 54 | 3 | 0 | 0 | 2 | 0 |
|  |  | 64 | 3 | 2 | 0 | 2 | 0 |
|  | 002 | 52 | 3 | 0 | 0 | 0 | 0 |
|  |  | 62 | 3 | 0 | 0 | 0 | 0 |
| 3 | 001 | 13 | 3 | 2 | 0 | 0 | 0 |
|  |  | 44 | 3 | 2 | 0 | 0 | 0 |
|  | 003 | 20 | 3 | 0 | 0 | 0 | 0 |
|  |  | 39 | 3 | 0 | 0 | 0 | 0 |

*Detailed Phenodex scoring in each organ system:

S3: lax and redundant skin; E1, peau d’orange; E2: angioid streaks; C0: no cardiac symptoms or history of ischemic heart disease; V0: no symptoms or signs of arterial insufficiency; V2: intermittent claudication.
